# Factors associated with the use of the N95 respirator in university students in the daily life of COVID-19

**DOI:** 10.1590/0034-7167-2021-0412

**Published:** 2022-07-18

**Authors:** Jhon Alex Zeladita Huaman, María Josefa Arcaya Moncada, Roberto Zegarra Chapoñan, Gilmer Solis Sánchez, Rosane Gonçalves Nitschke, Iris Jara Huayta

**Affiliations:** I Universidad Nacional Mayor de San Marcos. Lima, Peru; II Universidad María Auxiliadora. Lima, Peru; III Universidad Científica del Sur. Lima, Peru; IV Universidade Federal de Santa Catarina. Florianópolis, Santa Catarina, Brazil; V Universidad Nacional de San Cristóbal de Huamanga. Ayacucho, Peru

**Keywords:** Students, Nursing, COVID-19, Age Factors, Security Measures, Activities of Daily Living., Estudantes de Enfermagem, Infecções por Coronavírus, Fatores de Idade, Medidas de Segurança, Atividades Cotidianas., Estudiantes de Enfermería, Infecciones por Coronavirus, Factores de Edad, Medidas de Seguridad, Actividades Cotidianas.

## Abstract

**Objective:**

To identify the factors associated with the use of the N95 respirator in Nursing and Medical students in the daily life of the covid-19 pandemic.

**Methods:**

Descriptive-analytical cross-sectional study carried out in 2020. A total of 830 students from three universities in Peru participated in the study. Associations were evaluated using Pearson’s Chi-Square and multivariate Poisson modeling with log linkage.

**Results:**

Statistically significant differences were found between the preference to use the N95 respirator in relation to masks according to the activity they perform (p=0.001) and where they live (p=0.005). The multivariate analysis reported that the associated characteristics were age, activity performed, perception and fear of being infected by covid-19.

**Conclusion:**

The choice of N95 respirator is influenced by individual factors and perceptions. Spaces are needed to discuss daily life, the way of living, caring and educating, considering the socioeconomic dimensions and beliefs.

## RESUMEN

**Objetivo:**

Identificar los factores asociados al uso del respirador N95 en estudiantes de Enfermería y Medicina Humana en lo cotidiano de la pandemia COVID-19.

**Métodos:**

Estudio descriptivo-analítico transversal realizado en el año 2020. Participaron 830 estudiantes de tres universidades de Perú. Se evaluaron asociaciones mediante Chi Cuadrado de Pearson y modelamiento multivariado de Poisson con link log.

**Resultados:**

Se encontró diferencias estadísticamente significativas entre la preferencia de usar el respirador N95 en comparación con las mascarillas según la actividad que desempeñan (p=0,001) y lugar de residencia (p=0,005). El análisis multivariado reportó que las características asociadas fueron la edad, la actividad que desempeña, percepción y miedo de que podrían infectarse con COVID-19.

**Conclusión:**

En la preferencia de elegir el respirador N95, intervienen factores individuales y las percepciones. Se necesitan espacios para discutir lo cotidiano, la manera de vivir, cuidar y educar; considerando dimensiones socioeconómicas y creencias.

## RESUMO

**Objetivo:**

Identificar os fatores associados ao uso do respirador N95 em estudantes de Enfermagem e Medicina no cotidiano da pandemia de COVID-19.

**Métodos:**

Estudo descritivo-analítico transversal, realizado em 2020. Participaram 830 estudantes de três universidades do Peru. As associações foram avaliadas por meio do Qui-Quadrado de Pearson e modelagem multivariada de Poisson com link de log.

**Resultados:**

Foram encontradas diferenças estatisticamente significativas entre a preferência de uso do respirador N95 em relação às máscaras, de acordo com a atividade exercida (p=0,001) e o local de residência (p=0,005). A análise multivariada relatou que as características associadas foram a idade, a atividade que desempenha, a percepção e o medo de ser infectado pela COVID-19.

**Conclusão:**

Na preferência de escolha do respirador N95, intervêm fatores e percepções individuais. São necessários espaços para discutir o cotidiano, o modo de viver, cuidar e educar, considerando as dimensões socioeconômicas e as crenças.

## INTRODUCTION

The rapid spread of COVID-19 has had an impact on everyday and contemporary life. By May 2021, 155 million people had been diagnosed globally, of which 3.2 had died from this disease^(1)^. Meanwhile, Peru ranked fourth in the world with the highest mortality rate (192078 per 1000000 inhabitants)^(2)^ and faced the expansion of the second wave with an accumulated 1.8 million diagnosed cases and 62976 deaths^(3)^, even the Peruvian government having declared a state of health emergency and implemented socio-sanitary measures recommended by the World Health Organization (WHO), such as quarantine, suspension of face-to-face classes in educational institutions, distance education and mandatory use of protective measures^(4)^.

From the declaration of the COVID-19 pandemic, the WHO recommended the use of the mask as an individual protection measure to reduce contagion, along with other measures such as hand washing, physical distancing and the use of face mask^(5)^. At the beginning of the pandemic, there was controversy about the type of mask that the population should use. Subsequently, a secondary investigation of randomized trials showed that the use of N95 respirators significantly protects against clinical respiratory disease and infections compared with masks^(6)^. This is because these respirators can filter particles up to 0.3 μm, while surgical masks filter particles up to 5 μm^(7)^.

The decision to wear a mask is determined by multiple sociodemographic and community factors^(8)^. Studies carried out in viral pandemics indicate that there is a consensus that women, those who declare their marital status as married and those who live in urban areas, report greater use of masks^(9-10)^. However, there is a discrepancy between the association of age, education and income level with the use of these devices ^(11-12)^. Regarding beliefs and emotions, they agree that there is a positive correlation between concern, fear, stress, perception of susceptibility, severity and effectiveness with the use of masks^(9-10,13)^. At the same time, they point out that there is a contradiction with the perception of risk^(14-15)^. Finally, it was reported that knowledge about the disease is related to the use of masks^(12)^.

It is noteworthy that Nursing and Medicine students are a vulnerable group to be infected with COVID-19 due to factors such as caring for their families in the face of the collapse of the health system and for carrying out pre-professional practices in health establishments, especially in the last years of his university education. Knowing that there is little evidence on the aspects that determine the use of N95 respirators in university students, the need and relevance of carrying out this research was realized, since understanding the multifactorial influences of the use of masks is essential to design effective strategies that guarantee respiratory protection of Health Sciences students^(8)^.

This study will provide information on the aspects that determine the choice for the use of the N95 respirator in Health Sciences students. It evidences that it will allow the design of guidelines, intervention programs and educational strategies that promote the use of more effective respiratory devices for the control and prevention of COVID-19, especially in university students during daily life, understanding everyday life as “the way of life of the human being that is shown in everyday life, through their interactions, beliefs, values, meanings, culture, symbols, which outline their process of living, in a movement of being healthy and sick, directing its life cycle”^(16)^.

## OBJECTIVE

To identify the factors associated with the use of the N95 respirator in Nursing and Medical students in the daily life of the COVID-19 pandemic.

## METHODS

### Ethical aspects

The study was approved by the Research Ethics Committee of the Medical course from the Universidad Nacional Mayor de San Marcos (draft number 030). In addition, the informed consent form was applied to all participants.

### Type of study

Quantitative, descriptive-analytical and cross-sectional study, guided according to the STROBE tool, carried out between July and October 2020, in three public universities in Peru located in different natural regions. Universidad San Cristóbal de Huamanga is located in the Ayacucho region (cordillera), the Universidad Nacional Toribio Rodríguez de Mendoza, in the Amazon region (jungle) and the Universidad Nacional San Marcos, in Lima (coast). This manuscript reports the analysis of factors associated with the use of the N95 respirator, which is part of a study carried out with students of Health Sciences on preventive measures against COVID-19^(17)^.

### Population and sample: inclusion and exclusion criteria

The population consisted of 2596 Health Sciences students, 1061 of them in Nursing and 1508 in Medicine. The inclusion criteria were: being a student in any of the indicated careers, being enrolled in the 2020-I semester and agreeing to participate in the study. Students under 18 years old and those who did not have digital electronic equipment such as a computer, laptop or cell phone with internet access were excluded. After having access to the entire population, information was obtained from 830 students. The type of sampling was non-probabilistic for convenience.

### Study protocol

Prior to data collection, a document for authorization request was sent to the deans of the Health Sciences courses of the selected universities. Subsequently, a meeting was held with each of the directors of the professional schools to report the study and request their authorization to distribute the form among the students.

The technique used was the survey through a self-administered questionnaire, prepared in Google Forms, disseminated among students through emails and social networks. The content and construct of the instrument were validated by health professionals, specialists working in the areas of Epidemiology, methodologists, and members of the COVID command of each of the regions where the study was conducted. In addition, a pilot test was carried out on 30 students, 10 from each university. The information collected in this validation process was excluded from the data analysis.

The questionnaire was structured in three sections: in the first one, the study and informed consent were described. In the second one, the possible associated factors were addressed. In the third one, they were asked about the main type of mask they preferred to use: cloth mask, surgical mask and N95 respirators (NIOSH N95 or KN95), the frequency of use and care with these devices (washing cloth masks and circumstances exchange of surgical masks).

The possible associated factors considered in the study were: sex, age group, marital status, activity performed, if you have or had a family member or acquaintance diagnosed with COVID, if any family member died from this disease, course, place where they leave, perception that they could get infected, fear of getting infected and susceptibility to getting infected with COVID-19.

### Data analysis

For statistical analysis, Stata version 16.1 (Stata Corporation, College Station, Texas, USA) was used. Quantitative variables were described as mean, standard deviation, median and interquartile range, and qualitative variables expressed as frequencies and proportions.

A descriptive evaluation of the variables collected for sample characterization was performed. Then, the students’ behavior regarding the use and care of the masks were evaluated. The evaluation of the characteristics associated with the type of mask used was performed using Pearson’s Chi-Square test to calculate the P value. The factors associated with the use of the N95 respirator were evaluated by the generalized linear model (GLM) of the Poisson family, with log link function. The model adjustment was performed including those variables that in the raw model presented p-value < 0.200, as defined by Bursac et al^(18)^. Crude and adjusted prevalence ratios (PR) were reported in the models, with 95% confidence intervals. Inferential analyzes were performed using a significance level of 0.05.

## RESULTS

A total of 830 students from the three universities were enrolled. 47.3% (392) of them studied at the Universidad Nacional de San Cristóbal de Huamanga, 27.3% (227) at the Universidad Nacional Mayor de San Marcos and 25.4% (211) at the Universidad Nacional Toribio Rodríguez de Mendoza. Table 1 shows the sociodemographic characteristics of Health Sciences students, mostly women (72.7%), aged between 20 and 29 years old (76.4%) and single marital status (96.9%). It is noteworthy that 24.1% of students or a member of their family were diagnosed with COVID-19.

**Table 1:** Sociodemographic characteristics of students, 2020

**Characteristic**	**n**	**%**
Sex		
Male	226	27.3
Female	604	72.7
Age group		
Under 20 years old	182	21.9
From 20 to 29 years old	634	76.4
30 years old or older	14	1.7
Marital status		
Single	804	96.9
Married and living together	26	3.1
Activity performed		
Dedicated only to studies	541	65.2
Studying and working	289	34.8
The student or any member of his/her family has been diagnosed with COVID-19		
No	630	75.9
Yes	200	24.1
Any family member died of COVID-19		
No	761	91.7
Yes	69	8.3
Study career		
Nursing	585	70.5
Medicine	245	29.5
Place where she/he lives		
Rural area	177	21.3
Urban area	653	78.7

### Perceptions about COVID-19

Regarding the students’ perceptions (table 2), 46.6% consider that they could not become infected with COVID-19. However, 37.0% reported being afraid of becoming infected with COVID-19 and 50.6% considered themselves somewhat susceptible to catching this disease.

**Table 2:** Perceptions of students about COVID-19, 2020

**Perceptions**	**n**	**%**
Thinks she/he could get infected with COVID-19		
Definitively no	70	8.4
No	387	46.6
Yes	291	35.1
Absolutely yes	82	9.9
Afraid of getting infected with COVID-19		
Definitively no	101	12.2
No	236	28.4
Yes	307	37.0
Absolutely yes	186	22.4
Considers to be susceptible to contagion by COVID-19		
Nada susceptible	23	2.7
Poco susceptible	266	32.1
Algo susceptible	420	50.6
Muy susceptible	121	14.6

### Use and management of respiratory protection measures

Regarding respiratory protection (Table 3), more than half of the interviewees reported that they mainly use the surgical mask (51.6%), while 26.1% use the N95 respirator. It is noteworthy that the frequency of use that predominates in all respiratory protection devices is always. Regarding handling, students wash the cloth mask daily (65.4%) and one in two students change the surgical mask after each use.

**Table 3:** Behaviors regarding the use and handling of masks and N95 respirator of the surveyed students, 2020

**Conduct**	**n**	**%**
Main type of mask used		
Fabric mask	217	26.1
Surgical mask	428	51.6
N95 respirator	185	22.3
Total	830	100.0
When leaving the house, how often do you use the cloth mask?		
Sometimes	15	6.9
Most of the time	79	36.4
Always	123	56.7
Total	217	100.0
How often do you wash your cloth mask?		
Daily	142	65.4
2 or more days	75	34.6
Total	217	100.0
How often do you wash your cloth mask?		
Sometimes	30	7.0
Most of the times	61	14.2
Always	337	78.8
Total	428	100.00
Under what circumstances do you change your surgical mask?		
After each use	214	50.0
When it is dirty	113	26.4
When it is wet	81	18.9
When it breaks	20	4.7
Total	428	100.0
When leaving home, how often do you use the N95 respirator?		
Sometimes	3	1.6
Most of the times	30	16.2
Always	152	82.2
Total	185	100.0

### Characteristics associated with the use of the N95 respirator

Bivariate analysis (Table 4) reported that N95 respirator use was higher among students who only study (77.6%) than among students who study and work (66.8%). Similarly, among students living in urban areas (76.1%) compared to students living in rural areas such as population centers (65.5%). Both differences were statistically significant. The other variables evaluated were not characteristics associated with the use of the N95 respirator.

**Table 4:** Characteristics associated with the type of mask used mainly during the COVID-19 pandemic by the students surveyed (bivariate analysis)

**Sociodemographic** **characteristics and** **perceptions**	**Uses fabric or surgical mask** **n (%)**	**Uses N95 respirator** **n (%)**	*p* value^*^
Sex			
Male	54 (23.9)	172 (76.1)	0.367
Female	163 (27.0)	441 (73.0)	
Age group			
Under 20 years old	41 (22.5)	141 (77.5)	0.103
From 20 to 29 years old	175 (27.6)	459 (72.4)	
30 years old or older	1 (7.1)	13 (92.9)	
Marital status			
Single	211 (26.2)	593 (73.8)	0.718
Married and living together	6 (23.1)	20 (76.9)	
Activity performed			
Dedicated only to studies	121 (22.4)	420 (77.6)	0.001
Studying and working	96 (33.2)	193 (66.8)	
The student or any member of his/her family has been diagnosed with COVID-19			
No	175 (27.8)	455 (72.2)	0.057
Yes	42 (21.0)	158 (79.0)	
Has any family member died of COVID-19			
No	204 (26.8)	557 (73.2)	0.149
Yes	13 (18.8%)	56 (81.2)	
Study career			
Nursing	162 (27.7)	423 (72.3)	0.117
Medicine	55 (22.5)	190 (77.5)	
Place where she/he lives			
Countryside	61 (34.5)	116 (65.5)	0.005
Urban area	156 (23.9)	497 (76.1)	
Think she/he can get infected with COVID-19			
Definitely not	22 (31.4)	48 (68.6)	0.366
No	107 (27.7)	280 (72.3)	
Yes	71 (24.4)	220 (75.6)	
Absolutely yes	17 (20.7)	65 (79.3)	
Afraid of getting infected with COVID-19			
Definitely not	21 (20.8)	80 (79.2)	0.398
No	59 (25.0)	177 (75.0)	
Yes	89 (29.0)	218 (71.0)	
Absolutely yes	48 (25.8)	138 (74.2)	
Considers to be susceptible to contagion by COVID-19			
Not at all susceptible	4 (17.4)	19 (82.6)	0.705
Little susceptible	74 (27.8)	192 (72.2)	
Susceptible	107 (25.5)	313 (74.5)	
Very susceptible	32 (26.5)	89 (73.5)	
Total	217 (26.1)	613 (73.9)	

* Pearson’s chi-square.

Regarding the multivariate analysis (Table 5), the crude model reported that the variables associated with the use of the N95 respirator were age (P=0.032), the activity performed (P=0.002), which the student or household member diagnosed with COVID-19 (P=0.042) and place where they live (P=0.011). However, the adjusted analysis reported age (p=0.015), the activity they perform (P=0.003), the perception that one could be infected with COVID-19 (P=0.016) and the fear of becoming infected with COVID -19 (P=0.015) were characteristics associated with the use of the N95 respirator. Students over 30 years old are 26% more likely (PR 1.26, 95%CI 1.05 - 1.52) to use the N95 respirator compared to students under 20 years old. Students who work are 4% less likely (PR 0.86, 95%CI 0.78 - 0.95) to use the N95 respirator compared to students who only study.

**Table 5:** Multivariate analysis of factors associated with N95 respirator use during the COVID-19 pandemic by students surveyed

**Sociodemographic characteristics** **and perceptions**	**Crude**		**Adjusted**	
	**PR (95 %CI)**	***p* value**	**PR (95 %CI)**	***p* value**
Sex				
Male	Reference		----	----
Female	0.96 (0.88 - 1.05)	0.354	----	----
Age group				
Under 20 years old	Reference		Reference	
From 20 to 29 years old	0.93 (0.85 - 1.02)	0.149	0.94 (0.86 - 1.03)	0.191
30 years or older	1.20 (1.02 - 1.41)	0.032	1.26 (1.05 - 1.52)	0.015
Marital status				
Single	Reference		----	----
Married and living together	1.04 (0.84 - 1.29)	0.701	----	----
Activity performed				
Dedicated only to studies	Reference		Reference	
Studying and working	0.86 (0.78 - 0.94)	0.002	0.86 (0.78 - 0.95)	0.003
The student or any member of his/her family has been diagnosed with COVID-19				
No	Reference		Reference	
Yes	1.09 (1.00 - 1.19)	0.042	1.05 (0.96 - 1.15)	0.265
Any family member died of COVID-19				
No	Reference		Reference	
Yes	1.11 (0.98 - 1.25)	0.096	1.09 (0.97 - 1.24)	0.159
Study career				
Nursing	Reference		Reference	
Medicine	1.07 (0.99 - 1.17)	0.103	1.02 (0.93 - 1.11)	0.677
Place where they live				
Rural area	Reference		Reference	
Urban area	1.16 (1.03 - 1.30)	0.011	1.09 (0.97 - 1.23)	0.144
Think she/he can get infected with COVID-19				
Definitely not	Reference		Reference	
No	1.06 (0.89 - 1.25)	0.537	1.15 (0.95 - 1.38)	0.143
Yes	1.10 (0.93 - 1.31)	0.265	1.20 (0.99 - 1.46)	0.062
Absolutely yes	1.16 (0.95 - 1.40)	0.142	1.30 (1.05 - 1.61)	0.016
Afraid of getting infected with COVID-19				
Definitely not	Reference		Reference	
No	0.95 (0.84 - 1.07)	0.389	0.92 (0.81 - 1.04)	0.189
Yes	0.90 (0.79 - 1.01)	0.081	0.85 (0.75 - 0.97)	0.015
Absolutely yes	0.94 (0.82 - 1.07)	0.328	0.91 (0.79 - 1.04)	0.171
Considers to be susceptible to contagion by COVID-19				
Not at all susceptible	Reference		Reference	
Little susceptible	0.87 (0.71 - 1.07)	0.190	0.85 (0.67 - 1.07)	0.169
Susceptible	0.90 (0.74 - 1.10)	0.303	0.85 (0.68 - 1.08)	0.186
Very susceptible	0.89 (0.72 - 1.11)	0.292	0.82 (0.64 - 1.05)	0.114

## DISCUSSION

Daily, Health Sciences students have several respiratory protection devices such as cloth masks, surgical masks or N95 respirators. According to the WHO recommendation, they should be used along with other preventive measures to prevent and avoid the transmission of COVID-19^(19)^. Compliance with these measures was mandatory due to regulations enacted by the Peruvian government. Thus, the research was carried out with Nursing and Medicine students who did not start clinical practice by order of the National Superintendence of Higher Education. The main finding was that the preference for using an N95 respirator was associated with age, activity they perform, perception of the likelihood that they could be infected and fear of COVID-19.

One of the theories used in the analysis of the factors that determine the adoption of preventive behaviors and risk perception of COVID-19 is the health belief model^(20)^. Based on this theoretical model, the perception of the risk to which one is exposed or has been exposed may suppose another conditioning factor for behavior. Thus, the finding of this study shows, in the daily life of the COVID-19 pandemic, that students who are afraid of becoming infected and those who are afraid of this disease are more likely to choose and use the N95 respirator than surgical or cloth face masks.

This finding suggests that students who perceive themselves to be more susceptible to contracting the disease adopt more effective prevention measures in their daily lives. Aspect evidenced in a study carried out in the context of the Severe Acute Respiratory Syndrome pandemic, since the use of the mask was associated with the perception of risk of contracting the disease^(14)^. Another recent study, carried out in ten countries, found that there is an association between risk perception and the practices of preventive measures for COVID-19^(15)^. Likewise, in Brazil, it has been reported that people who practice physical activity at the recommended levels are less likely to wear a mask when leaving home^(21)^ and that the adoption of protective measures in people with multiple comorbidities was higher compared to people without comorbidity^(22)^.

The finding that students under 20 years old are less likely to choose the N95 respirator compared to those over 30 could be explained by the invulnerability bias in which people, based on their personal characteristics, attribute who will have more favorable results compared to other people^(23)^. Thus, a qualitative study reported that the feeling of invincibility is a barrier to the use of N95 respirators in health professionals who work in the care of patients affected by tuberculosis^(24)^. It is noteworthy that age was reported as a predictor of mask use^(10)^ and that the percentage of people who wear a mask is higher among people over 50 years old than among people of other ages^(11)^.

In the study, it is reported that working students are less likely to wear the N95 respirator compared to those who only study. In this regard, previous studies reported that the use of masks is greater in the unemployed population in relation to those who perform some work activity^(11-12)^. This finding shows that those who work and study live in a situation of greater vulnerability, as they cannot buy a more expensive product such as the N95 respirator because they cannot afford it^(25)^.

In other words, the subjective value will be the desire to avoid illness and the expectation will be the belief that a possible action will reduce the risk of getting sick. Thus, it is suggested to carry out studies that evaluate other aspects such as the cost of the devices, the type of activity they perform or the type of transport they use.

During August 2020, Peru was in the first wave of COVID-19^(3)^, infections, during which time data collection began. Thus, it was found that approximately one in five students preferred to use the N95 respirator, a percentage higher than that reported by a study carried out with nursing students in Turkey^(13)^. This would indicate that students of Health Sciences are at risk of contracting this disease because, generally, they are the ones who take care of family members with health problems, even more if we consider that the infection rate derived from a primary case in a Peruvian household was 53%^(26)^.

Another finding of the study is that most students report that they mainly used surgical masks, while in the general population the use of fabric masks predominates^(27)^. One of the reasons that may explain this difference is that Health Science students know how the different types of masks work, their effectiveness and the protection they provide. It is important to emphasize that the evidence on the use of surgical masks is heterogeneous and of low quality. Likewise, it was not possible to determine its effectiveness in reducing the risk of transmission of respiratory diseases caused by viruses. On the other hand, there is no evidence on the effectiveness of using cloth masks as a protection factor^(6)^.

### Limitations of the study

The study had some limitations. Among them, it can be mentioned that the choice of a non-probabilistic sample does not allow the results to be generalized to other contexts. Furthermore, in the study, other variables such as self-efficacy, which could affect the results, were not considered because, in the analysis of the adoption of prevention measures, in addition to risk, self-efficacy intervenes. Likewise, as the collection was carried out through an online questionnaire, the information should be considered as self-report, which can be affected by bias. Finally, it is one of the first studies that explores the preference for the use of the N95 respirator, as no antecedents were found to allow comparison of results.

### Contributions to the area of Nursing

Countries face a regrettable and worrying situation due to the high transmission of COVID-19 among the population and the number of deaths. This situation requires the joint effort of health institutions and academics to promote more effective respiratory protection practices, in order to prevent contagion, as this disease is transmitted mainly through the respiratory route^(19)^. The everyday factors of the COVID-19 pandemic reported in this study need to be considered to design educational strategies and interventions to promote the use of N95 respirators, especially among Health Science students who, when resuming their clinical practices, must acquire these devices. We also have to take into account that, in Peru, health professionals without an employment relationship receive the N95 respirator from the health unit less frequently to rethink their way of living and caring for themselves^(28)^.

## CONCLUSIONS

In this study carried out with Nursing and Medicine students from three universities in Peru, it was identified that the factors associated with the preference for the use of the N95 respirator over the use of the mask are predominantly at the individual level, such as age, activity they perform and perception of who may be infected and the fear of COVID-19. Regarding the perceptions that Health Sciences students have, most of them do not consider that they can become infected but are afraid of becoming infected. They realize that they are somewhat susceptible to being infected by COVID-19. Regarding the use of personal protective devices, most use mainly surgical masks on a regular basis.

The findings on the preference for the use of the N95 respirator by students of Health Sciences suggest that the adoption of more effective respiratory protection measures would be associated with socioeconomic factors, risk perception and fear.

This study promotes important contributions in the prevention of COVID-19 in Health Sciences students, as it brings new scientific evidence on the influence of individual factors on the decision to use the N95 respirator, an information that can serve as support to guide the teaching and design of norms or intervention programs that promote the prevention of COVID-19 in universities. Spaces are needed where people can stop and discuss everyday life, their way of living, caring and educating, considering socioeconomic dimensions and perceptions.

## SUPLEMENTARY MATERIAL

To request information about the questionnaire or other study information, you must send an email to the corresponding author.
